# A phase II study of cisplatin and 5-fluorouracil with concurrent hyperfractionated thoracic radiation for locally advanced non-small-cell lung cancer: a preliminary report from the Okayama Lung Cancer Study Group

**DOI:** 10.1054/bjoc.1999.0885

**Published:** 1999-12-08

**Authors:** Y Segawa, H Ueoka, K Kiura, H Kamei, M Tabata, K Sakae, Y Hiraki, S Kawahara, K Eguchi, S Hiraki, Mine Harada for the Okayama Lung Cancer Study Group

**Affiliations:** Department of Internal Medicine, National Shikoku Cancer Center Hospital, 13 Horinouchi, Matsuyama, 790-0007, Japan; Second Department of Internal Medicine, Department of Radiology, Okayama University Medical School, 2-5-1 Shikata-cho, Okayama, 700-8558, Japan; Department of Internal Medicine, National South Okayama Hospital, 4066 Hayashima-cho, Tsukubo-gun, Okayama, 701-0304, Japan; Department of Internal Medicine, Okayama Red Cross Hospital, 2-1-1 Aoe, Okayama, 700-8607, Japan

**Keywords:** combined chemoradiotherapy, hyperfractionated thoracic radiation, cisplatin, 5-fluorouracil, non-small-cell lung cancer

## Abstract

A recent meta-analysis and randomized studies have demonstrated that combined chemoradiotherapy is associated with a survival advantage for selected patients with locally advanced unresectable non-small-cell lung cancer (NSCLC). We conducted a phase II study of combined chemoradiotherapy to find a more effective combination of drugs and radiation than those previously reported for such patients. Between January 1994 and November 1996, 50 previously untreated patients with locally advanced unresectable NSCLC (stage IIIA with N2 or IIIB disease) were entered in this study. Patients were required to have Eastern Cooperative Oncology Group performance status ≤ 2, age ≤ 75 years and adequate organ function. Treatment consisted of three cycles of cisplatin (20 mg m^−2^, days 1–5) and 5-fluorouracil (5-FU) (500 mg m^−2^, days 1–5) every 4 weeks, and concurrent hyperfractionated thoracic radiation (1.25 Gy twice daily, with a 6-h interfraction interval; total radiation dose, 62.5–70 Gy). Of the 50 patients entered, 37 (74%) responded to this chemoradiotherapy, including two (4%) with complete response. By a median follow-up time of 41.0 months, 35 patiennts had died and 15 were stil alive. The median time to progression for responding patients was 14.1 months (range, 2.6–51.3+ months). The median survival time was 18.7 months, with a survival rate of 66.0% at 1 year, 46.0% at 2 years and 27.6% at 3 years. Survival outcome was strongly affected by the extent of nodal involvement (median survival time, 27.4 months for N0–2 disease (*n* = 37) vs 10.7 months for N3 disease (*n* = 13);*P* = 0.007). The major toxicities of treatment were leukopenia and neutropenia (≥ Grade 3, 58% and 60% respectively). Other toxicities of ≥ Grade 3 included thrombocytopenia (26%), anaemia (26%), nausea/vomiting (16%) and radiation oesophagitis (6%). Treatment-related death occurred for one patient. Our findings suggest that cisplatin and 5-FU in combination with concurrent hyperfractionated thoracic radiation is effective and feasible for the treatment of locally advanced unresectable NSCLC. The short-term survival in this study appeared to be more encouraging than those of similar chemoradiation trials. A randomized trial will be needed to compare the combination of cisplatin and 5-FU with other platinum-based regimens together with concurrent hyperfractionated thoracic radiation. In addition, in future studies, inclusion criteria for N3 disease with or without supraclavicular involvement should be reconsidered to correctly evaluate the effect of combined chemoradiotherapy for locally advanced unresectable NSCLC. © 2000 Cancer Research Campaign


*British Journal of Cancer* (2000) **82**(1), 104–111
© 2000 Cancer Research Campaign
Article no. bjoc.1999.0885

### A phase II study of cisplatin and 5-fluorouracil with concurrent hyperfractionated thoracic radiation for locally advanced non-small-cell lung cancer: a preliminary report from the Okayama Lung Cancer Study Group

**Y Segawa[1], H Ueoka[2], K Kiura[2], H Kamei[2], M Tabata[2], K Sakae[3], Y Hiraki[3], S Kawahara[4], K Eguchi[1], S Hiraki[5] and M Harada[2] for the Okayama Lung Cancer Study Group**

[1]Department of Internal Medicine, National Shikoku Cancer Center Hospital, 13 Horinouchi, Matsuyama 790-0007, Japan; [2]Second Department of Internal Medicine and [3]Department of Radiology, Okayama University Medical School, 2-5-1 Shikata-cho, Okayama 700-8558, Japan; [4]Department of Internal Medicine, National South Okayama Hospital, 4066 Hayashima-cho, Tsukubo-gun, Okayama 701-0304, Japan; [5]Department of Internal Medicine, Okayama Red Cross Hospital, 2-1-1 Aoe, Okayama 700-8607, Japan

**Summary** A recent meta-analysis and randomized studies have demonstrated that combined chemoradiotherapy is associated with a survival advantage for selected patients with locally advanced unresectable non-small-cell lung cancer (NSCLC). We conducted a phase II study of combined chemoradiotherapy to find a more effective combination of drugs and radiation than those previously reported for such patients. Between January 1994 and November 1996, 50 previously untreated patients with locally advanced unresectable NSCLC (stage IIIA with N2 or IIIB disease) were entered in this study. Patients were required to have Eastern Cooperative Oncology Group performance status ≤ 2, age ≤ 75 years and adequate organ function. Treatment consisted of three cycles of cisplatin (20 mg $m^{-2}$, days 1–5) and 5-fluorouracil (5-FU) (500 mg $m^{-2}$, days 1–5) every 4 weeks, and concurrent hyperfractionated thoracic radiation (1.25 Gy twice daily, with a 6-h interfraction interval; total radiation dose, 62.5–70 Gy). Of the 50 patients entered, 37 (74%) responded to this chemoradiotherapy, including two (4%) with complete response. By a median follow-up time of 41.0 months, 35 patients had died and 15 were still alive. The median time to progression for responding patients was 14.1 months (range, 2.6–51.3+ months). The median survival time was 18.7 months, with a survival rate of 66.0% at 1 year, 46.0% at 2 years and 27.6% at 3 years. Survival outcome was strongly affected by the extent of nodal involvement (median survival time, 27.4 months for N0–2 disease ($n$ = 37) vs 10.7 months for N3 disease ($n$ = 13); $P$ = 0.007). The major toxicities of treatment were leukopenia and neutropenia (≥ Grade 3, 58% and 60% respectively). Other toxicities of ≥ Grade 3 included thrombocytopenia (26%), anaemia (26%), nausea/vomiting (16%) and radiation oesophagitis (6%). Treatment-related death occurred for one patient. Our findings suggest that cisplatin and 5-FU in combination with concurrent hyperfractionated thoracic radiation is effective and feasible for the treatment of locally advanced unresectable NSCLC. The short-term survival in this study appeared to be more encouraging than those of similar chemoradiation trials. A randomized trial will be needed to compare the combination of cisplatin and 5-FU with other platinum-based regimens together with concurrent hyperfractionated thoracic radiation. In addition, in future studies, inclusion criteria for N3 disease with or without supraclavicular involvement should be reconsidered to correctly evaluate the effect of combined chemoradiotherapy for locally advanced unresectable NSCLC. © 2000 Cancer Research Campaign

**Keywords:** combined chemoradiotherapy; hyperfractionated thoracic radiation; cisplatin; 5-fluorouracil; non-small-cell lung cancer

For patients with non-small-cell lung cancer (NSCLC), surgical resection offers the best chance for long-term survival. However, approximately two-thirds of NSCLC patients have unresectable disease. Although radiotherapy has played an important role in the treatment of locally advanced unresectable NSCLC, the treatment outcome for such patients has remained poor due to both locoregional and systemic failures (median survival time (MST), 9.1 months; 2-year survival rate, 13.5%) (Gould et al, 1995). To improve this disappointing situation, many approaches have been tried, including a modified fractionation method for radiation (Cox et al, 1990; Saunders et al, 1996), combined chemoradiotherapy (Le Chevalier et al, 1991; Blanke et al, 1995; Furuse et al, 1995; Jeremic et al, 1995, 1996; Sause et al, 1995; Dillman et al, 1996; Lee et al, 1996), and preoperative chemoradiotherapy (Weiden et al, 1991; Strauss et al, 1992; Albain et al, 1995; Choi et al, 1997; Eberhardt et al, 1998). Among these modalities of treatment, survival advantage has been demonstrated for patients receiving combined chemoradiotherapy (Jeremic et al, 1995, 1996; Sause et al, 1995; Dillman et al, 1996). In a meta-analysis of 22 randomized clinical trials (chemoradiotherapy vs radiotherapy alone), chemoradiotherapy resulted in a 10% reduction in the annual risk of death and a consequent improvement in 2-year survival from 15% to 18% (Non-small Cell Lung Cancer Collaborative Group, 1995). In addition, cisplatin-based chemotherapy yielded better

*Received 26 January 1999*
*Revised 11 May 1999*
*Accepted 8 July 1999*

*Correspondence to:* Y Segawa results than those obtained with other drugs and their combinations (Non-small Cell Lung Cancer Collaborative Group, 1995).

In this study, we employed a combination of cisplatin and 5-fluorouracil (5-FU) demonstrated to have synergistic antitumour activity in both preclinical (Scanlon et al, 1986; Esaki et al, 1992) and clinical studies (Rooney et al, 1985; Kemeny et al, 1990). For lung cancer, this combination yielded a response rate ranging from 25% to 47% (Lynch et al, 1994; Gemma et al, 1995; Tsuchiya et al, 1995), although 5-FU alone is thought to be inactive against NSCLC (response rate, 8.1%) (Citron et al, 1992). The mechanism of synergism between these two drugs remains unclear. However, there are various hypotheses concerning the modulatory effect of cisplatin on 5-FU, and vice versa; concerning the former, it has been suggested that cisplatin-induced increase of reduced intracellular folate level potentiates the effect of 5-fluorodeoxyuridine monophosphate by forming a covalent ternary complex with thymidylate synthase, leading to enhanced 5-FU cytotoxicity (Scanlon et al, 1986), while concerning the latter, it has been suggested that modulation of cisplatin-induced DNA-adduct repair by 5-FU results in enhanced cisplatin cytotoxicity (Esaki et al, 1992).

Hyperfractionated radiation therapy is expected to increase antitumour effects and decrease toxicity to normal tissues (Cox et al, 1990; Roach et al, 1995; Segawa et al, 1997). In a published series, patients receiving hyperfractionated radiation therapy appeared to have a better prognosis than those treated with conventional radiotherapy (Sause et al, 1995; Bonner et al, 1998). Considering the radiosensitizing effects of cisplatin and 5-FU (Vokes et al, 1990), it appears possible that concurrent combination of hyperfractionated radiation therapy with these drugs will increase their antitumour effect by increasing the frequency of interaction between chemotherapy and radiotherapy.

Based on this background, the Okayama Lung Cancer Study Group conducted a prospective phase II study to evaluate the efficacy and toxicity of a combination of cisplatin and 5-FU combined with concurrent hyperfractionated thoracic radiation for locally advanced unresectable NSCLC.

### PATIENTS AND METHODS

### Eligibility criteria

This phase II study was designed to treat locally advanced, surgically unresectable NSCLC. Based on the TNM staging system adopted in 1986 (Mountain, 1986), patients with stage IIIA with N2 or IIIB disease were eligible for inclusion in this study. Patients with malignant pleural or pericardial effusion, or with pleural dissemination were excluded. Patients were required to have a histologically or cytologically confirmed diagnosis of NSCLC, previously untreated disease, measurable lesions, Eastern Cooperative Oncology Group (ECOG) performance status (PS) ≤ 2, age ≤ 75 years and no history of malignancy within 5 years before enrolment. Any patients who had previously undergone chemotherapy or radiotherapy were excluded from this study.

Before enrolment, each patient gave a complete medical history and underwent physical, laboratory and staging work-up examinations. Laboratory examinations included complete blood cell counts, serum chemistry and tumour marker analyses, 24-h creatinine clearance evaluation, arterial blood gas analysis, urinalysis, electrocardiogram and pulmonary function tests. Staging work-up examination consisted of chest plain radiographs, computerized tomography (CT) of the chest and abdomen (ultrasonography of the abdomen could be substituted), magnetic resonance imaging of the brain, radionucleotide bone scan and fibreoptic bronchoscopy. Mediastinoscopy was not included in the staging work-up examination. On laboratory examination, patients were required to have a white blood cell (WBC) count ≥ 4000 $\mu l^{-1}$, platelet (PLT) count ≥ 100 000 $\mu l^{-1}$, haemoglobin level ≥ 9 g $dl^{-1}$, serum bilirubin level ≤ 1.5 mg $dl^{-1}$, serum AST and ALT levels ≤ 2.5 times the upper limit of normal, 24-h creatinine clearance level ≥ 60 ml $min^{-1}$ and arterial oxygen pressure ≥ 60 mmHg. Patients with markedly diminished pulmonary function status (i.e. ≤ 50% of the predicted vital capacity and/or ≤ 40% forced expiratory volume in 1 s of the predicted value), and those with serious complications such as unstable angina, congestive heart failure, uncontrolled diabetes mellitus, or severe obstructive pneumonia were excluded from this study.

Written informed consent was obtained from all patients. Four institutions participated in this study, and their Institutional Review Boards approved this study. The patients were entered in this study after verification of eligibility by the central registration office (Second Department of Internal Medicine, Okayama University Medical School).

### Chemotherapy

Eligible patients received three cycles of chemotherapy consisting of cisplatin (20 mg $m^{-2}$, days 1–5) and 5-FU (500 mg $m^{-2}$, days 1–5) every 4 weeks. Cisplatin was intravenously administered for 30 min, followed by 2-h infusion of 5-FU. Before and after cisplatin instillation, all patients received 2000–2500 ml infusion over 4 h. In the second and third cycles of chemotherapy, dose modification was performed based on the haematological toxicities observed in the prior cycle of chemotherapy. Modified doses of cisplatin and 5-FU were as follows: 24 mg $m^{-2}$ and 600 mg $m^{-2}$ at dose level +1, 16 mg $m^{-2}$ and 400 mg $m^{-2}$ at dose level –1, and 14 mg $m^{-2}$ and 300 mg $m^{-2}$ at dose level –2. The two drugs were administered at dose level +1 when leukopenia or neutropenia remained at ≤ Grade 2, without thrombocytopenia; they were administered at dose level –1 when Grade 4 leukopenia or neutropenia, or Grade 3 or higher thrombocytopenia developed; they were administered at dose level –2 when severe infection or bleeding tendency associated with haematological toxicity was observed. In case of acceptable toxicities other than those mentioned above, chemotherapy was repeated at the starting dose. In addition, at the time of the next cycle of chemotherapy, the drugs were administered at dose level –1 when WBC and PLT counts were 3000–3900 $\mu l^{-1}$ and 75 000–99 000 $\mu l^{-1}$ respectively. Chemotherapy was not given until haematological recovery when WBC and PLT counts were less than 3000 $\mu l^{-1}$ and 75 000 $\mu l^{-1}$ respectively. The cisplatin dose was reduced by half when 24-h creatinine clearance level decreased below 60 ml $min^{-1}$ but was ≥ 30 ml $min^{-1}$. Neither drug was given when 24-h creatinine clearance level was less than 30 ml $min^{-1}$.

All patients received prophylactic antiemetic therapy using 5-hydroxytryptamine type 3 receptor blocker and/or dexamethasone. When Grade 3 or higher leukopenia or neutropenia occurred, recombinant human granulocyte colony stimulating factor (*r*hG-CSF) administration was permitted under the guidelines of the Japanese Ministry of Health and Welfare.

© 2000 Cancer Research Campaign

### Radiotherapy

Radiotherapy was started on day 1 of chemotherapy, using a linear accelerator (6–10 MeV). The radiation dose was 2.5 Gy with two fractions per day (1.25 Gy per fraction) with a 6-h interfraction interval. A total dose of 62.5–70 Gy was delivered in 10 fractions per week for 5 consecutive days. The treatment volumes consisted of original and boost volumes. The initial dose of 45 Gy was administered to the original volume, which included the site of primary tumour with a margin of 2 cm around the mass and the ipsilateral hilum, and the whole width of the mediastinum with a margin of 1 cm around the radiographically visible region of involvement extending inferiorly to 3 cm below the carina or 2 cm below the radiographically-demonstrated tumour mass. The supraclavicular region was further included if involved with tumour. An additional 17.5–25 Gy was administered to the boost volume that included the site of primary tumour and involved lymph nodes. The original volume was treated with an anterior-posterior parallel-opposed pair of portals, and the boost volume was treated with the same pair or with a pair of oblique fields if cumulative radiation dose to the spinal cord was over 45 Gy.

When Grade 3 or higher radiation oesophagitis toxicity occurred, radiation therapy was withheld until radiation oesophagitis recovered to ≤ Grade 2. On the other hand, in case of Grade 3 or higher leukopenia or neutropenia, radiation therapy was not discontinued.

### Response and toxicity evaluation

For evaluation of response and toxicity, all patients treated on an inpatient basis underwent a series of examinations consisting of complete blood cell counts, serum chemistry and plain chest radiographs on at least a weekly basis during the treatment period and then on a monthly basis. CT scans of the chest were performed after each cycle of chemotherapy, and the same examinations as for the staging work-up study were performed after completion of the treatment.

Responses were assessed using the World Health Organization (WHO) criteria (Miller et al, 1981). The response to treatment, including eligibility and assessibility, was determined for each patient by extramural reviewers. Complete response (CR) was defined as the disappearance of all measurable lesions for at least 4 weeks. Partial response (PR) was defined as a ≥ 50% decrease in the sum of the products of the greatest perpendicular diameters of all measurable lesions for at least 4 weeks without the development of new lesions. Progressive disease (PD) was defined as a ≥ 25% increase in the sum of the products of the perpendicular diameters of all measurable disease or the appearance of new lesions. If no response or progression of disease occurred during therapy, treatment outcome was considered no change (NC). Toxicities were assessed using the WHO criteria (Miller et al, 1981), and grading of acute oesophageal toxicity due to radiation was evaluated in accordance with that of oral toxicity.

### Statistical analysis

The sample size of this study was determined on the assumption that the expected response rate was 75%, with a 95% confidence interval (CI) of ± 12.5%.

Statistical analyses were performed using the SPSS Base System™ and Advanced Statistics™ programs (SPSS Inc, Chicago, IL, USA). For comparison of proportions for categorical variables, the $\chi^2$ test was used. Survival time was defined as the period from the initiation of treatment to death or last follow-up evaluation. In addition, time to progression was defined as the period from the initiation of treatment to PD. Survival curves were calculated using the method of Kaplan and Meier, and differences in survival distribution between two categorized groups were assessed using a log-rank test. To estimate the prognostic significance of covariates for survival, a Cox regression model was employed in backward step-wise fashion. *P*-values less than 0.05 in two-tailed analyses were considered significant.

**Table 1** Patient characteristics

| Characteristic | No. of patients |
|---|---|
| No. of patients entered | 50 |
| No. of patients eligible | 50 |
| Median age in years (range) | 63 (38–74) |
| Gender | |
| Male | 45 |
| Female | 5 |
| ECOG performance status | |
| 0 | 17 |
| 1 | 30 |
| 2 | 3 |
| Weight loss | |
| < 10% | 33 |
| ≥ 10% | 17 |
| Histology | |
| Squamous cell carcinoma | 22 |
| Adenocarcinoma | 20 |
| Large-cell carcinoma | 6 |
| Adenosquamous cell carcinoma | 1 |
| Unclassified carcinoma | 1 |
| Stage of disease[a] | |
| IIIA | 13 |
| IIIB | 37 |
| TNM classification[a] | |
| T4N0M0 | 6 |
| T4N1M0 | 1 |
| T1N2M0 | 4 |
| T2N2M0 | 4 |
| T3N2M0 | 5 |
| T4N2M0 | 17 |
| T2N3M0 | 6 |
| T3N3M0 | 3 |
| T4N3M0 | 4 |

[a] TNM staging system adopted in 1986.

### RESULTS

### Patient characteristics

Between January 1994 and November 1996, 50 patients were entered in this study, and all were included for analyses of response, survival and toxicity. Patient characteristics are shown in Table 1. There were 45 males and five females, with a median age of 63 years (range 38–74 years). Forty-seven (94%) patients had a good ECOG PS of 0–1, and 17 (34%) had weight loss ≥ 10% during the 6-month period preceding the study entry. Twenty-two (44%) patients had squamous cell carcinoma, 20 (40%) adenocarcinoma, six (12%) large cell carcinoma, and one (2%) each adenosquamous cell carcinoma and unclassified carcinoma. Thirteen (26%) patients had stage IIIA and 37 (74%) stage IIIB

 © 2000 Cancer Research Campaign

**Table 2** Response rate and survival by pretreatment factors

| Variable | No. of patients | Response CR | PR | NC | PD | NE | Response rate (%) | P-value | MST (months) | P-value |
|---|---|---|---|---|---|---|---|---|---|---|
| Overall | 50 | 2 | 35 | 8 | 3 | 2 | 74.0 | | 18.7 | |
| Age | | | | | | | | | | |
| ≤ 63 years | 25 | 2 | 16 | 3 | 3 | 1 | 72.0 | | 24.0 | |
| > 63 years | 25 | 0 | 19 | 5 | 0 | 1 | 76.0 | 0.747 | 18.6 | 0.998 |
| Gender | | | | | | | | | | |
| Male | 45 | 2 | 30 | 8 | 3 | 2 | 71.1 | | 23.8 | |
| Female | 5 | 0 | 5 | 0 | 0 | 0 | 100.0 | 0.162 | 17.7 | 0.854 |
| ECOG performance status | | | | | | | | | | |
| 0–1 | 47 | 2 | 32 | 8 | 3 | 2 | 72.3 | | 24.0 | |
| 2 | 3 | 0 | 3 | 0 | 0 | 0 | 100.0 | 0.290 | 10.5 | 0.080 |
| Weight loss | | | | | | | | | | |
| < 10% | 33 | 2 | 22 | 6 | 2 | 1 | 72.7 | | 27.2 | |
| ≥ 10% | 17 | 0 | 13 | 2 | 1 | 1 | 76.5 | 0.775 | 16.5 | 0.254 |
| Histology | | | | | | | | | | |
| Adenocarcinoma | 20 | 0 | 12 | 5 | 2 | 1 | 60.0 | | 18.6 | |
| Non-adenocarcinoma | 30 | 2 | 23 | 3 | 1 | 1 | 83.3 | 0.065 | 18.7 | 0.308 |
| Stage of disease[a] | | | | | | | | | | |
| IIIA | 13 | 0 | 12 | 1 | 0 | 0 | 92.3 | | 32.7 | |
| IIIB | 37 | 2 | 23 | 7 | 3 | 2 | 67.6 | 0.080 | 16.5 | 0.186 |
| T factor[a] | | | | | | | | | | |
| 0–3 | 22 | 0 | 16 | 3 | 2 | 1 | 72.7 | | 24.0 | |
| 4 | 28 | 2 | 19 | 5 | 1 | 1 | 75.0 | 0.856 | 17.7 | 0.838 |
| N factor[a] | | | | | | | | | | |
| 0–2 | 37 | 2 | 29 | 5 | 0 | 1 | 83.8 | | 27.4 | |
| 3 | 13 | 0 | 6 | 3 | 3 | 1 | 46.2 | 0.008 | 10.7 | 0.007 |

[a] TNM staging system adopted in 1986. MST, median survival time; NE, not evaluable.

disease. The mediastinum, heart, great vessels, trachea, oesophagus, or vertebral body was involved in 28 (56%) patients. Based on the extent of nodal involvement, six (12%) patients had N0, one (2%) N1, 30 (60%) N2 and 13 (26%) N3 disease. Of patients with N3 disease, four (8%) had unilateral or bilateral metastasis to supraclavicular lymph nodes.

### Treatment accomplishment

Thirty-three (66%) patients completed three cycles of chemotherapy, with dose elevation for two (4%) and reduction for 11 (22%) patients. The second and third cycles of chemotherapy were not administered to six (12%) and 17 (34%) patients respectively. Reasons for not completing chemotherapy were patient refusal ($n$ = 5), toxicity or death ($n$ = 5), physician's discretion ($n$ = 4) and disease progression ($n$ = 3). Cases of discontinuation at physician's discretion included deterioration of PS for two patients, and cerebral infarction and massive haemoptysis requiring bronchial arterial embolization in one case each. Treatment-related death occurred for one patient, due to disseminated intravascular coagulation syndrome caused by infection associated with severe neutropenia. The median interval between the first and second cycles of chemotherapy was 32.5 days (range 21–70 days), while that between the second and third cycles was 35 days (25–51 days). The actuarial dose intensities of chemotherapy (administered dose per time unit/projected dose per time unit; mean ± standard deviation (s.d.) were 73.6 ± 16.3% for cisplatin and 73.4 ± 16.8% for 5-FU.

Forty-seven (94%) patients completed radiotherapy; however, six (12%) of the patients required a rest period from radiation (median, 15.5 days; range 11–43 days), due to radiation oesophagitis ($n$ = 3), deterioration of PS ($n$ = 2), or fever of unknown origin ($n$ = 1). Total radiation dose and duration (mean ± s.d.) were 68.5 ± 3.9 Gy and 43.6 ± 6.2 days respectively. In addition, of the 47 patients who completed radiotherapy, ten received a total radiation dose of 62.5–67.5 Gy while 37 received 70 Gy. Of 44 patients who received the second cycle of chemotherapy, 38 (76%) received it during the period of radiotherapy. Overall, 33 (66%) of the 50 patients completed both chemotherapy and radiotherapy with no or minor modification of dose or schedule. In addition, two (4%) patients who had a good response after the completion of this therapy underwent surgery, although this study was not intended to include an adjuvant surgery setting. Pathologic CR was confirmed in one of these patients, and both were still alive without recurrence of disease at the last follow-up evaluation (24.6 and 51.3 months respectively).

### Response

Responses to the combined chemoradiotherapy are summarized in Table 2. Of the 50 patients entered, two (4%) had CR, 35 (70%) PR, eight (16%) NC and three (6%) PD. The remaining two (4%) patients were suspended from response evaluation due to treatment-related death and cerebral infarction in one case each. Therefore, the overall response rate was 74%, with a 95% CI of 61.8–86.2%. There were no differences in response rate by age (≤ 63 vs > 63 years, $P$ = 0.747), gender ($P$ = 0.162), ECOG PS (0 to 1 vs 2, $P$ = 0.290), weight loss (< 10% vs ≥ 10%, $P$ = 0.775), or T factor group (T0–3 vs T4, $P$ = 0.856). However, patients with N0–2 disease responded significantly better than those with N3 disease (83.8% vs 46.2%; $P$ = 0.008). The difference in response rate was even more striking on comparison with a subgroup of

© 2000 Cancer Research Campaign 

**Table 3** Pattern of initial failure

| Initial recurrence | No. of patients | % |
|---|---|---|
| No. of patients evaluated[a] | 48 | |
| No. of patients failed | 34 | 70.8 |
| Local progression only | 20 | 41.7 |
| Distant progression only | 10 | 20.8 |
| Brain (+ liver) | 6 (1) | |
| Bone | 2 | |
| Adrenal | 1 | |
| Skin | 1 | |
| Local + distant progression | 4 | 8.3 |
| Retroperitoneal lymph node | 2 | |
| Cervical lymph node | 1 | |
| Liver | 1 | |

[a] Two patients who were suspended from response evaluation were excluded from this analysis.

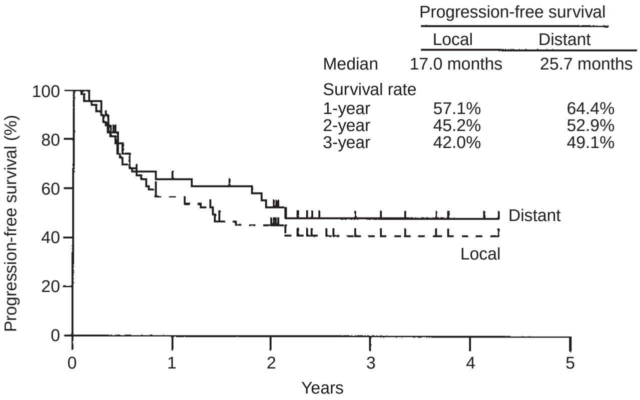

**Figure 1** Local and distant progression-free survival curves patients with supraclavicular lymph node metastasis (*n* = 4) (83.8% vs 0%, *P* < 0.001). In addition, non-adenocarcinoma patients and those with stage IIIA disease tended to respond better than those with adenocarcinoma (*P* = 0.065) and those with stage IIIB disease (*P* = 0.080) respectively. There were no significant differences in response rate between the other histological groups (e.g. 81.8% for squamous cell carcinoma vs 67.6% for non-squamous cell carcinoma, *P* = 0.264).

By a median follow-up time of 41.0 months (range 24.2–53.2 months), 25 of the 37 responding patients had relapsed. The median time to progression for the responding patients was 14.1 months (range 2.6–51.3+ months). Excluding the two patients suspended from response evaluation, the most common site of initial failure was local (*n* = 24, 50%) and the second most common site was brain (*n* = 6, 12.5%) (Table 3). The local progression-free survival rate was 57.1% at 1 year, 45.2% at 2 years and 42.0% at 3 years, with a median of 17.0 months (Figure 1). In addition, the distant progression-free survival rate was 64.4% at 1 year, 52.9% at 2 years and 49.1% at 3 years, with a median of 25.7 months (Figure 1).

### Survival

The survival curve for the 50 patients is shown in Figure 2. By a median follow-up time of 41.0 months, 35 (70%) patients had died

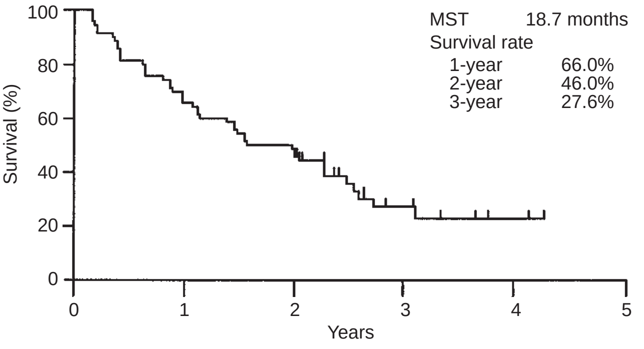

**Figure 2** Survival curve. MST, median survival time and 15 (30%) were still alive. The cause of death was directly related to NSCLC for 33 patients and unrelated for two (treatment-related death and pulmonary infarction in one case each). The survival rate was 66.0% at 1 year, 46.0% at 2 years and 27.6% at 3 years, with an MST of 18.7 months. There were no differences in survival by age (≤ 63 vs > 63 years, *P* = 0.998), gender (*P* = 0.854), weight loss (< 10% vs ≥ 10%, *P* = 0.254), histology (adenocarcinoma vs non-adenocarcinoma, *P* = 0.308), disease stage (IIIA vs IIIB, *P* = 0.186), or T factor group (T0–3 vs T4, *P* = 0.838) (Table 2). However, patients with N0–2 disease had significantly better survival than those with N3 disease (MST, 27.4 vs 10.7 months, *P* = 0.007). Similar to the findings for response rates, the difference in survival was strongly significant on comparison with the subgroup of patients with supraclavicular lymph node metastasis (MST, 27.4 vs 2.1 months, *P* < 0.001). In addition, patients with a good ECOG PS of 0–1 tended to have better survival than those with PS of 2 (MST, 24.0 vs 10.5 months, *P* = 0.080).

Factors influencing survival were further assessed using a Cox regression model. All of the parameters listed in Table 2 were included and analysed in backward step-wise fashion. The finally selected model ($\chi^2$ (1) = 7.381, *P* = 0.007) demonstrated that N factor was a dominant prognostic factor in our series of NSCLC patients (hazard ratio, 2.653; 95% CI 1.278–5.505; *P* = 0.009).

### Toxicity

Toxicities observed in the 50 patients during treatment and the follow-up period are listed in Table 4. The major toxicity was myelosuppression. Grade 3 or higher leukopenia and neutropenia occurred in 29 (58%) and 30 (60%) patients respectively. In addition, Grade 3 or higher thrombocytopenia and anaemia occurred in 13 (26%) patients each. *r*hG-CSF was administered following 35 (28.7%) of the 122 assessable cycles of chemotherapy, for a median duration of 6 days (range 2–24 days). In addition, 15 patients underwent *r*hG-CSF administration during radiotherapy while 35 did not. Grade 3 or higher thrombocytopenia was observed in four of the former 15 patients (PLT nadir counts: 17 000, 21 000, 40 000 and 44 000 $\mu l^{-1}$), with a median duration of 4 days (range 3–12 days), although ≥ Grade 3 thrombocytopenia was observed in none of the remaining 35 patients. Non-haematologic toxicities were generally mild, although Grade 3 or higher radiation eosophagitis occurred in three (6%) patients. No Grade 3 or higher radiation pneumonitis was observed in this study. Overall, ≥ Grade 3 toxicity was observed in 37 (74%) patients; 34 (68%) of them experienced haematological toxicity alone,

 *© 2000 Cancer Research Campaign*

**Table 4** Haematological and non-haematological toxicities

| Toxicity | No. of patients with toxicities | | | | % of toxicities |
|---|---|---|---|---|---|
| | Grade 1 | Grade 2 | Grade 3 | Grade 4 | ≥ Grade 3 |
| Leukopenia | 3 | 18 | 22 | 7 | 58 |
| Neutropenia | 8 | 11 | 18 | 12 | 60 |
| Thrombocytopenia | 5 | 10 | 5 | 8 | 26 |
| Anaemia | 7 | 18 | 10 | 3 | 26 |
| Nausea/vomiting | 20 | 13 | 8 | 0 | 16 |
| Diarrhoea | 6 | 0 | 1 | 0 | 2 |
| Fever | 8 | 7 | 0 | 0 | 0 |
| Alopecia | 24 | 10 | 0 | 0 | 0 |
| Renal dysfunction | 3 | 2 | 0 | 0 | 0 |
| Liver dysfunction | 3 | 1 | 0 | 0 | 0 |
| Peripheral neurotoxicity | 2 | 0 | 0 | 0 | 0 |
| Oesophagitis | 22 | 6 | 1 | 2 | 6 |
| Pneumonitis | 13 | 4 | 0 | 0 | 0 |
| Worst haematological toxicity per patient | 2 | 14 | 18 | 16 | 68 |
| Worst non-haematological toxicity per patient | 12 | 26 | 7 | 2 | 18 |
| Worst toxicity per patient | 1 | 12 | 20 | 17 | 74 |

while nine (18%) of them experienced only nonhaematologic toxicity.

### DISCUSSION

Combined chemoradiotherapy is now accepted as a standard form of therapy for locally advanced unresectable NSCLC, based on results of both a recent meta-analysis (Non-small Cell Lung Cancer Collaborative Group, 1995) and large-scaled randomized trials (Sause et al, 1995; Dillman et al, 1996) that compared combined chemoradiotherapy with radiotherapy alone. Accordingly, the interest of many investigators is focused on the timing and mode of radiotherapy, and on the selection and combination of drugs for patients with this condition. Although possible advantage of concurrent combination of chemotherapy and radiotherapy over sequential combination was suggested in a West Japan Lung Cancer Group trial (Furuse et al, 1997), definitive conclusions have not been obtained concerning this issue, including whether fractionated radiation methods such as standard fractionation or hyperfractionation are useful. In this study, the effectiveness and feasibility of cisplatin and 5-FU in combination with concurrent hyperfractionated thoracic radiation were evaluated to find a promising concurrent chemoradiotherapy regimen for locally advanced unresectable NSCLC.

Regarding treatment outcome in this study, 66% of the patients completed both chemotherapy and radiotherapy with no or only minor modification of the treatment schedule. The overall response rate and MST were 74% and 18.7 months respectively. In addition, both locoregional and distant progression-free survivals were fairly good (median values, 17.0 and 25.7 months respectively). The major toxicities were leukopenia and neutropenia, for which *r*hG-CSF was administered following 28.7% of the chemotherapy cycles. Although *r*hG-CSF was administered to 30% of the patients during radiotherapy following the first cycle of chemotherapy, life-threatening thrombocytopenia was a rare event within the period of radiotherapy. The incidences of Grade 3 or higher radiation oesophagitis and pneumonitis were quite low (6% and 0% respectively), in contrast to those in a series of concurrent chemoradiotherapy trials (12–53% and 1–25% respectively) (Lee et al, 1996; Blanke et al, 1997; Choy et al, 1998; Jeremic et al, 1998; Clamon et al, 1999). Additionally, although this study used different boost dose settings (17.5–25 Gy), no significant differences were found in response rate, survival, or toxicity profile between the different boost dose groups (data not shown).

Results of concurrent chemoradiotherapy trials in a published series and our own are listed in Table 5. For a phase III series, Jeremic et al (1995, 1996) reported significantly favourable survival in each arm consisting of weekly or daily carboplatin/etoposide combined with hyperfractionated thoracic radiation compared with this type of radiotherapy-alone arm. In a trial reported by Clamon et al (1999), 10% lower local recurrence rate was demonstrated for induction chemotherapy followed by concurrent chemoradiotherapy arm than for the sequential chemoradiotherapy arm, but this improvement did not lead to a survival advantage. Interestingly, Bonner et al (1998) found a possible survival advantage for hyperfractionated radiation therapy over standard radiotherapy in a subset analysis of their study. In a phase II series, several platinum-based chemotherapy regimens were concurrently combined with standard or hyperfractionated thoracic radiotherapy with differing dose settings for radiation. Among these, Jeremic et al (1998) and Choy et al (1998) reported promising treatment results in terms of MST (25 and 20.5 months respectively). In addition, the 2-year and 3-year survival rates (46.0% and 27.6% respectively) in our trial were among the most encouraging obtained.

However, our findings should be carefully interpreted due to the small sample size and non-randomized setting. In general, treatment outcome is strongly affected by the clinical background of patients enroled. This study included only a few patients with poor PS, but included some ineligible patients with weight loss ≥ 10% and those with N3 disease. Patients with N3 disease in this study had extremely poor survival compared with those with N0–2 disease (MST, 10.7 vs 27.4 months), although the diagnosis of N3 disease was made based mainly on chest CT imaging, with a criterion of short-axis diameter of lymph node ≥ 1 cm. This difference in survival was even more striking when compared with the survival of the subgroup of patients with supraclavicular lymph node metastasis (MST, 2.1 vs 27.4 months). Given the large proportion of patients with weight loss, a well-known indicator of poor prognosis for NSCLC (Paesmans et al, 1995), this study does

© 2000 Cancer Research Campaign 

**Table 5** Phase III and II studies of concurrent chemoradiotherapy for locally advanced unresectable non-small-cell lung cancer

| First author | RT (Gy) | Concurrent chemotherapy | No. of patients | MST (months) | Survival rate (%) 1-year | 2-year | 3-year | P-value |
|---|---|---|---|---|---|---|---|---|
| Phase III study | | | | | | | | |
| Blanke (1995) | 60–65 Std | | 111 | 10.6 | 45 | 13 | 3 | |
| | 60–65 Std | CDDP | 104 | 9.9 | 43 | 18 | 9 | 0.35 |
| Jeremic (1995) | 64.8 Hfx | | 61 | 8 | 39 | 25 | 6.6 | |
| | 64.8 Hfx | weekly CBDCA+ETP | 52 | 18 | 73 | 35 | 23 | 0.027[c] |
| | 64.8 Hfx | biweekly CBDCA+ETP | 56 | 13 | 50 | 27 | 16 | 0.17[c] |
| Jeremic (1996) | 69.6 Hfx | | 66 | 14 | 68 | 26 | 11 | |
| | 69.6 Hfx | daily CBDCA+ETP | 65 | 22 | 74 | 43 | 23 | 0.021 |
| Bonner (1998) | 60 Std | | 34 | 8.6 | – | – | – | |
| | 60 Hfx | | 33 ] | | | | | |
| | 60 Hfx | CDDP+ETP | 32 ] | 11.6 | – | – | – | 0.10 |
| Clamon (1999)[a] | 60 Std | | 137 | 13.5 | 54 | 26 | 19 | |
| | 60 Std | weekly CBDCA | 146 | 13.4 | 56 | 29 | 19 | 0.743 |
| Phase II study | | | | | | | | |
| Furuse (1995) | 50–60 Std | CDDP+VDS+MMC | 61 | 16 | 60 | 36.7 | 23.1 | |
| Lee (1996) | 69.6 Hfx | CDDP+ETP | 76 | 18.9 | 67 | 35 | – | |
| Blanke (1997) | 60.4 Std | daily CDDP+ETP | 20 | 11.6 | 45 | – | – | |
| Jeremic (1998) | 69.6 Hfx | daily CBDCA+ETP | 41 | 25 | 83 | 51 | 34 | |
| Lau (1998)[b] | 61 Std | CBDCA+ETP | 60 | 13 | – | 21 | – | |
| Choy (1998) | 66 Std | weekly CBDCA+TXL | 39 | 20.5 | 56.3 | 38.3 | – | |
| Present study | 62.5–70 Hfx | CDDP+5-FU | 50 | 18.7 | 66.0 | 46.0 | 27.6 | |

RT, radiotherapy: Hfx, hyperfractionation; Std, standard fractionation. Abbreviations for chemotherapy drugs: CBDCA, carboplatin; CDDP, cisplatin; ETP, etoposide; 5-FU, 5-fluorouracil; MMC, mitomycin C; TXL, taxol; VDS, vindesine; VLB, vinblastine. MST, median survival time. [a]All the patients received induction chemotherapy consisting of cisplatin and vinblastine, followed by thoracic radiotherapy in one arm and concurrent chemoradiotherapy in the other arm. [b]This study was conducted on a series of poor-risk patients. [c]Compared with radiotherapy alone arm.

not appear to have a strong bias in patient selection yielding falsely positive treatment outcome.

Trials of preoperative chemotherapy and radiotherapy for stage IIIA with N2 and/or IIIB disease reported MSTs ranging from 13 to 25 months (Weiden et al, 1991; Strauss et al, 1992; Albain et al, 1995; Choi et al, 1997; Eberhardt et al, 1998). Among these, Choi et al (1997) and Eberhardt et al (1998) have recently reported promising long-term survival with the use of concurrent chemotherapy and hyperfractionated radiation therapy. It is difficult to compare treatment outcomes between trials including and not including surgery because of their differences in patient eligibility. However, these results also indicate the effectiveness of concurrent chemotherapy and hyperfractionated radiation therapy for locally advanced unresectable NSCLC.

In the present study, we employed a combination of cisplatin and 5-FU. However, only a few findings are available concerning which types of drugs are well tolerated and provide synergistic effects with radiotherapy. It will thus be necessary to find effective drugs and their combinations, including new drugs such as taxans and topoisomerase I inhibitors.

In conclusion, a combination of cisplatin and 5-FU together with concurrent hyperfractionated thoracic radiation was found to be effective and feasible for the treatment of locally advanced unresectable NSCLC. A randomized trial will be needed to compare a combination of cisplatin and 5-FU with other platinum-based regimens together with concurrent hyperfractionated thoracic radiation. In addition, in future studies, inclusion criteria for N3 disease with or without supraclavicular involvement should be reconsidered to correctly evaluate the effect of combined chemoradiotherapy for locally advanced unresectable NSCLC.

### ACKNOWLEDGEMENTS

We thank Drs Masaaki Kataoka (National Shikoku Cancer Center Hospital), Noriko Kanzaki (National South Okayama Hospital), and Hidehiro Hayashi (Okayama Red Cross Hospital) for their collaboration as radiotherapists. We also thank Drs Rika Tabata and Ichiro Takata (Second Department of Internal Medicine, Okayama University Medical School) for collection and management of data.

### REFERENCES

Albain KS, Rusch VW, Crowley JJ, Rice TW, Turrisi AT, Weick JK, Lonchyna VA, Presant CA, McKenna RJ, Gandara DR, Fosmire H, Taylor SA, Stelzer KJ, Beasley KR and Livingston RB (1995) Concurrent cisplatin/etoposide plus chest radiotherapy followed by surgery for stage IIIA(N2) and IIIB non-small-cell lung cancer: mature results of Southwest Oncology Group phase II study 8805. *J Clin Oncol* **13**: 1880–1892

Bonner JA, McGinnis WL, Stella PJ, Marschke RF, Sloan JF, Shaw EG, Mailliard JA, Creagan ET, Ahuja RK and Johnson PA (1998) The possible advantage of hyperfractionated thoracic radiotherapy in the treatment of locally advanced nonsmall cell lung carcinoma. *Cancer* **82**: 1037–1048

Blanke C, Ansari R, Mantravadi R, Gonin R, Tokars R, Fisher W, Pennington K, O'Connor T, Rynard S, Miller M and Einhorn L (1995) Phase III trial of thoracic irradiation with or without cisplatin for locally advanced unresectable non-small-cell lung cancer: a Hoosier Oncology Group Protocol. *J Clin Oncol* **13**: 1425–1429

Blanke C, DeVore R, Shyr Y, Epstein B, Murray M, Hande K, Stewart S and Johnson D (1997) A pilot study of protracted low dose cisplatin and etoposide with concurrent thoracic radiotherapy in unresectable stage III nonsmall cell lung cancer. *Int J Radiat Oncol Biol Phys* **17**: 111–116

Choi NC, Carey RW, Daly W, Mathisen D, Wain J, Wright C, Lynch T, Grossbard M and Grillo H (1997) Potential impact on survival of improved tumor

 *© 2000 Cancer Research Campaign*

downstaging and resection rate by preoperative twice-daily radiation and concurrent chemotherapy in stage IIIA non-small-cell lung cancer. *J Clin Oncol* **15**: 712–722

Choy H, Akerley W, Safran H, Graziano S, Chung C, Williams T, Cole B and Kennedy T (1998) Multiinstitutional phase II trial of paclitaxel, carboplatin, and concurrent radiation therapy for locally advanced non-small-cell lung cancer. *J Clin Oncol* **16**: 3316–3322

Citron ML, Modeas C, Propert K, Goutsou M and Green MR (1992) Phase II trial of high-dose 24-hour continuous intravenous 5-fluorouracil for advanced non-small cell lung cancer. *Cancer Invest* **10**: 215–219

Clamon G, Herndon J, Cooper R, Chang AY, Rosenman J and Green MR (1999) Radiosensitization with carboplatin for patients with unresectable stage III non-small-cell lung cancer: a phase III trial of the Cancer and Leukemia Group B and the Eastern Cooperative Oncology Group. *J Clin Oncol* **17**: 4–11

Cox JD, Azarnia N, Byhardt RW, Shin KY, Emami B and Pajak TF (1990) A randomized phase I/II trial of hyperfractionated radiation therapy with total doses of 60.0 Gy to 79.2 Gy: possible survival benefit with ≥ 69.6 Gy in favorable patients with Radiation Therapy Oncology Group stage III non-small-cell lung carcinoma: report of Radiation Therapy Oncology Group 83–11. *J Clin Oncol* **8**: 1543–1555

Dillman RO, Herndon J, Seagren SL, Eaton WL and Green MR (1996) Improved survival in stage III non-small-cell lung cancer: seven-year follow-up of Cancer and Leukemia Group B (CALGB) 8433 trial. *J Natl Cancer Inst* **88**: 1210–1215

Eberhardt W, Wilke H, Stamatis G, Stuschke M, Harstrick A, Menker H, Krause B, Müeller MR, Stahl M, Flasshove M, Budach V, Greschuchna D, Konietzko N, Sack H and Seeber S (1998) Preoperative chemotherapy followed by concurrent chemoradiation therapy based on hyperfractionated accelerated radiotherapy and definitive surgery in locally advanced non-small-cell lung cancer: mature results of a phase II trial. *J Clin Oncol* **16**: 622–634

Esaki T, Nakano S, Tatsumoto T, Kuroki-Migita M, Mitsugi K, Nakamura M and Niho Y (1992) Inhibition by 5-fluorouracil of *cis*-diamminedichloroplatinum(II)-induced DNA interstrand cross-link removal in a HST-1 human squamous carcinoma cell line. *Cancer Res* **52**: 6501–6506

Furuse K, Kubota K, Kawahara M, Kodama N, Ogawara M, Akira M, Nakajima S, Takada M, Kusunoki Y, Negoro S, Matsui K, Masuda N, Takifiji N, Kudoh S, Nishioka M and Fukuoka M (1995) Phase II study of concurrent radiotherapy and chemotherapy for unresectable stage III non-small-cell lung cancer. *J Clin Oncol* **13**: 869–875

Furuse K, Fukuoka M, Takada Y, Nishikawa H, Katagami N and Ariyoshi Y (1997) A randomized phase III study of concurrent versus sequential thoracic radiotherapy in combination with mitomycin, vindesine, and cisplatin in unresectable stage III non-small cell lung cancer: preliminary analysis. *Proc Am Soc Clin Oncol* **16**: 459a

Gemma A, Kudoh S, Yoshimura A, Ono Y, Takenaka K, Hayashihara K, Hino M, Shibuya M and Niitani H (1995) Pilot trial of a combination comprising of consecutive oral administration of UFT, and two-divided administration of CDDP in non-small cell lung cancer. *Anticancer Res* **15**: 2691–2696

Gould C and Sause WT (1995) Radiation therapy for lung cancer. In *Lung Cancer*, Johnson BE and Johnson DH (eds), pp. 191–207. Wiley-Liss: New York

Jeremic B, Shibamoto Y, Acimovic L and Djuric L (1995) Randomized trial of hyperfractionated radiation therapy with or without concurrent chemotherapy for stage III non-small-cell lung cancer. *J Clin Oncol* **13**: 452–458

Jeremic B, Shibamoto Y, Acimovic L and Milisavljevic S (1996) Hyperfractionated radiation therapy with or without concurrent low-dose daily carboplatin/etoposide for stage III non-small-cell lung cancer: a randomized study. *J Clin Oncol* **14**: 1065–1070

Jeremic B, Shibamoto Y, Milicic B, Nikolic N, Dagovic A and Milisavljevic S (1998) Concurrent radiochemotherapy for patients with stage III non-small-cell lung cancer (NSCLC): long-term results of a phase II study. *Int J Radiat Oncol Biol Phys* **42**: 1091–1096

Kemeny N, Israel K, Niedzwiecki D, Chapman D, Botet J, Minsky B, Vinciguerra V, Rosenbluth R, Bosselli R, Cochran C and Sheehan K (1990) Randomized study of continuous infusion fluorouracil versus fluorouracil plus cisplatin in patients with metastatic colorectal cancer. *J Clin Oncol* **8**: 313–318

Lau DH, Crowley JJ, Gandara DR, Hazuka MB, Albain KS, Leigh B, Fletcher WS, Lanier KS, Keiser WL and Livingston RB (1998) Southwest Oncology Group phase II trial of concurrent carboplatin, etoposide, and radiation for poor-risk stage III non-small-cell lung cancer. *J Clin Oncol* **16**: 3078–3081

Le Chevalier T, Arriagada R, Quoix E, Ruffie P, Martin M, Tarayre M, Lacombe-Terrier M, Douillard J and Laplanche A (1991) Radiotherapy alone versus combined chemotherapy and radiotherapy in nonresectable non-small-cell lung cancer: first analysis of a randomized trial in 353 patients. *J Natl Cancer Inst* **83**: 417–423

Lee JS, Scott C, Komaki R, Fossella FV, Dundas GS, McDonald S, Byhardt RW and Curran WJ (1996) Concurrent chemoradiation therapy with oral etoposide and cisplatin for locally advanced inoperable non-small-cell lung cancer: Radiation Therapy Oncology Group protocol 91–96. *J Clin Oncol* **14**: 1055–1064

Lynch TJ, Kalish LA, Kass F, Strauss G, Elias A, Skarin A, Shulman L, Sugarbaker D, and Frei E (1994) Continuous-infusion cisplatin, 5-fluorouracil, and leucovorin for advanced non-small cell lung cancer. *Cancer* **73**: 1171–1176

Miller AB, Hoogstraten B, Staquet M and Winkler A (1981) Reporting results of cancer treatment. *Cancer* **47**: 207–214

Mountain CF (1986) A new international staging system for lung cancer. *Chest* **89**: 225S–233S

Non-small Cell Lung Cancer Collaborative Group (1995) Chemotherapy in non-small cell lung cancer: a meta-analysis using updated data on individual patients from 52 randomized trials. *Br Med J* **311**: 899–909

Paesmans M, Sculier JP, Libert P, Bureau G, Dabouis G, Thiriaux J, Michel J, Van Custem O, Sergysels R, Mommen P and Klastersky J (1995) Prognostic factors for survival in advanced non-small-cell lung cancer: univariate and multivariate analyses including recursive partitioning and amalgamation algorithms in 1,052 patients. *J Clin Oncol* **13**: 1221–1230

Roach M, Gandara DR, Yuo H, Swift PS, Kroll S, Shrieve DC, Wara WM, Margolis L and Phillips TL (1995) Radiation pneumonitis following combined modality therapy for lung cancer: analysis of prognostic factors. *J Clin Oncol* **13**: 2606–2612

Rooney M, Kish J, Jacobs J, Kinzie J, Weaver A, Crissman J and Al-Sarraf H (1985) Improved complete response rate and survival in advanced head and neck cancer after three-course induction therapy with 120-hour 5-FU infusion and cisplatin. *Cancer* **55**: 1123–1128

Saunders MI, Dische S, Barrett A, Parmar MKB, Harvey A and Gibson D (1996) Randomized multicentre trials of CHART vs conventional radiotherapy in head and neck and non-small-cell lung cancer: an interim analysis. *Br J Cancer* **73**: 1455–1462

Sause WT, Scott C, Taylor S, Johnson D, Livingston R, Komaki R, Emami B, Curran WJ, Byhardt RW, Turrisi AT, Dar AR and Cox JD (1995) Radiation Therapy Oncology Group (RTOG) 88–08 and Eastern Cooperative Oncology Group (ECOG) 4588: preliminary results of a phase III trial in regionally advanced, unresectable non-small-cell lung cancer. *J Natl Cancer Inst* **87**: 198–205

Scanlon KJ, Newman EM, Lu Y and Priest DG (1986) Biochemical basis for cisplatin and 5-fluorouracil synergism in human ovarian carcinoma cells. *Proc Natl Acad Sci USA* **83**: 8923–8925

Segawa Y, Takigawa N, Kataoka M, Takata I, Fujimoto N and Ueoka H (1997) Risk factors for development of radiation pneumonitis following radiation therapy with or without chemotherapy for lung cancer. *Int J Radiat Oncol Biol Phys* **39**: 91–98

Strauss GM, Herndon JE, Sherman DD, Mathisen DJ, Carey RB, Choi NC, Rege VB, Modeas C and Green MR (1992) Neoadjuvant chemotherapy and radiotherapy followed by surgery in stage IIIA non-small-cell carcinoma of the lung: report of a Cancer and Leukemia Group B phase II study. *J Clin Oncol* **10**: 1237–1244

Tsuchiya S, Minato K, Nakano H, Takise A, Ezawa K, Fueki N, Hoshino H, Naruse I, Takei Y, Makimoto T, Nomoto T, Ishihara S, Mori M and Saito R (1995) A phase II study of 72-hour continuous infusion consisting of cisplatin and 5-fluorouracil for treatment of non-small cell lung cancer. *Oncology* **52**: 246–250

Vokes EE and Weichselbaum RR (1990) Concomitant chemoradiotherapy: rationale and clinical experience in patients with solid tumors. *J Clin Oncol* **8**: 911–934

Weiden PL and Piantadosi S (1991) Preoperative chemotherapy (cisplatin and fluorouracil) and radiation therapy in stage III non-small-cell lung cancer: a phase II study of the Lung Cancer Study Group. *J Natl Cancer Inst* **83**: 266–272

*© 2000 Cancer Research Campaign*
